# Changing Food Consumption Pattern and Influencing Factors in Bangladesh

**DOI:** 10.3390/foods12020401

**Published:** 2023-01-14

**Authors:** Mengmeng Jia, Lin Zhen, Wanni Yang, Shuang Wang

**Affiliations:** 1 College of Tourism, Henan Normal University, Xinxiang 453007, China; 2 Institute of Geographic Science and Natural Resources Research, Chinese Academy of Sciences, Beijing 100101, China; 3 University of Chinese Academy of Sciences, Beijing 100049, China; 4 China Center for Agricultural Policy, School of Advanced Agricultural Sciences, Peking University, Beijing 100871, China

**Keywords:** food consumption characteristic, food consumption pattern, influencing factor, Bangladesh

## Abstract

Food consumption is an important bridge between human beings and the natural ecosystem. The change in food consumption quantity and quality can reflect the relationship between them. This study aims to explore food consumption characteristics and the drivers of food consumption patterns in Bangladesh with a fragile ecology and polluted environment. This research selected food consumption in Bangladesh as the object, food consumption data were obtained from the Food and Agriculture Organization of the United Nations, and the data of influencing factors mainly were acquired from the World Bank. The following results were conducted: The total and per capita food consumption showed increase as a whole, but per capita food consumption experienced decline in the middle of the research period. Food consumption patterns were divided into three types: the first type of cereal–sugar–aquatic with low food consumption quantity and few kinds of food from 1961 to 1971, the second type of cereal–sugar–oil–aquatic with increasing food consumption quantity and food kinds from 1972 to 1997, and the third type of cereal–aquatic–tuber–sugar–fruit–vegetable–meat with increasing food consumption quantity and more various kinds of food from 1998 to 2020. The characteristics of food consumption in different periods were influenced by a series of factors. The influence of economic factors was higher than other factors, relatively. According to this study, the characteristics of food consumption patterns and the relationship between food consumption and influencing factors can provide a scientific reference for the adjustment policy makers taking local food demand and natural resources conservation into consideration to achieve a sustainable development.

## 1. Introduction

The resources needed for human development are all from the nature, especially the food that is necessary for human living. The change of food consumption quantity and structure could show the situation of living quality and local economic development to some extent. Food consumption change can reflect the relationship between human beings and the ecosystem. This relationship also is affected by a series of influencing factors such as population increase, environmental pollution, resources destruction, and extreme weather [1,2,3,4]; these factors take much negative effect on food security. Long-term exploitation and overuse of resources and environment by human beings has led to increasingly fragile ecology, depletion of resources, and aggravation of environmental pollution, which has seriously affected the sustainable development of human beings and nature.

The Millennium Ecosystem Assessment (MA) research report showed that the excessive and disorderly exploitation of natural resources by human beings was one of the main reasons for 60% of global ecological degradation [5]. At present, many scholars have carried out related research on the food consumption of ecosystem services. Many researchers [6,7] believed that the consumption of ecosystem services refers to the consumption, utilization, and occupation of products and services provided by the ecosystem by human beings directly or indirectly, in which human activities mainly included production activities and life activities. Based on econometrics and other theoretical foundations, some researchers [8,9,10,11] constructed the evaluation system and simulation method of ecosystem services, built the theoretical analysis foundation and method framework of production-consumption-valuation of ecosystem services, studied the influence of natural and artificial ecosystems on ecosystem services, and quantitatively analyzed the relationship between ecosystem services and supply. Some papers studied the influence of the pattern changes of ecosystem service consumption on local land resources and water resources based on the village and regional scale [12,13,14]. In the aspect of food consumption, the characteristics of food consumption in ecosystems of river basins and grassland belts are studied mainly by means of investigation and participatory assessment [15,16,17], but this category of research mainly studied the service content and characteristics of a single ecosystem service type in some areas, which could not fully reflect the overall situation of ecosystem service consumption in the study area. At present, there are studies on the characteristics of ecosystem service consumption at the global scale or national scale in countries along the belt and road initiative or a specific country by using biomass consumption measurement method [18,19]. This type of research does not involve research on the consumption of ecosystem services at smaller scales, such as urban and rural areas, and cannot conduct in-depth research on the spatial distribution dynamics of ecosystem services.

Bangladesh has the highest population density in the world. Especially with the further increase in population, food self-sufficiency is facing greater challenges. It is urgent to the characteristics of food consumption in this country to find more useful scientific reference to make more sustainable development strategies. This research aims to explore the evolution principle of food consumption in different ecosystems during different periods, and deeply explore the driving factors of food consumption change. The research object is the food consumption of Bangladeshi residents. This research mainly calculated and analyzed the food consumption of residents obtained from different ecosystems which contained farmland, grassland, forest, and aquatic ecosystem over the past 60 years (from 1961 to 2020). The influencing factors of the characteristics of food consumption were discussed. At the same time, from the perspective of ecology, society, and economy, we quantitatively explored the impact of resource supply, population growth, and social and economic development on food consumption, and clarified the relationship between social and economic development and food consumption, with a view to providing an important empirical reference for further research on the relationship between people and the ecosystem.

## 2. Methods

### 2.1. Study Area

Bangladesh is located on the delta plain in the northeast of the South Asian subcontinent. Most areas are located in the subtropical monsoon climate zone, which is hot and humid and rainy. The climate here is suitable for rice, corn, and other crops. The total area of this country is 147,570 km^2^. The economy mainly focuses on developing the agriculture. Its total population is nearly 163.05 million (World Bank, 2019). Bangladesh is one of the countries with the fastest economic growth in South Asia. In 2019, its gross domestic product (GDP) was about USD 257.869 billion, which got an increase of 7.88% over the previous year and a record high growth rate [20]. The land use in Bangladesh is mainly in the station of predatory development, and the land fertility is declining [21]. At the same time, accompanied by a large number of immigrants, there is a phenomenon of abandonment, resulting in waste of land resources [22]. Bangladesh suffers from frequent natural disasters, such as floods, hurricanes, and droughts [23]. In recent years, Bangladesh has witnessed rapid urbanization and industrialization. Due to its extensive development mode, it has caused serious pollution and damage to the local ecological environment [24].

### 2.2. Data Resources

The data from 1961 to 2020 used to calculate the consumption of various ecosystems in this study were mainly obtained from the Food and Agriculture Organization of the United Nations (FAO) [25]. The main data of factors such as population, GDP, per capita GDP, cereal production, and per capita consumption expenditure were acquired from the World Bank [26].

### 2.3. Methods

#### 2.3.1. Categories of Food Consumption Indicators in Different Ecosystems

The food consumption measured in this study was mainly the consumption of ecosystem products, excluding the consumption of ecosystem intangible products. Therefore, the consumption in this research mainly involved in the subsequent text referred to the consumption of food products in the ecosystem. According to different sources of food supply, ecosystem consumption was divided into farmland, grassland, forest, and aquatic ecosystem consumption. The farmland ecosystem mainly provided cereal, sugar, tuber, fruit, and beans for local residents. The kinds of food produced from the grassland ecosystem were meat, milk, and egg. The various fruits consumed were acquired, from the forest ecosystem. Apart from the previous ecosystems, the aquatic ecosystem could supply fish, shrimp, and other aquatic products. The consumption categories of various ecosystems included in this study are shown in Table 1.

#### 2.3.2. Food Consumption Calculation in Different Ecosystems

The calculation of food consumption in each ecosystem first measured the consumption of various products (the part directly used by humans) provided by the four types of ecosystems, namely farmland, grassland, forest and aquatic ecosystem. Then, the consumption of these products by residents was converted into the biomass consumed by the ecosystem to provide these products. Take wheat as an example, what the residents directly consumed was the cereal of wheat. It was necessary to convert the amounts of cereals eaten by people into the biomass of wheat, including the part of straw and root. Then, the total biomass of four ecosystems of different kinds of food could be obtained by adding the biomass of these different foods according to the source of the ecosystem [14]. The specific calculation formula is shown as follows:

(1) $$CUPM_{i}=P_{i}+I_{i}-E_{i}$$

where $CUPM_{i}$ represents residents’ consumption of various food provided by ecosystem and $P_{i}$, $I_{i}$, and $E_{i}$, respectively, represent production, import, and export of the specific food.

(1) Food consumption calculation in farmland ecosystem

Farmland ecosystem consumption mainly came from the ecosystem consumption needed by farmland to provide cereal, vegetables, beans, sugar, oil crops, and other products (Table 1). The specific calculation formulas are shown as follows [18,19]:

(2) $$CUEM_{A}=CUEM_{A1}+CUEM_{A2}$$

(3) $$CUEM_{A1}=\frac{CUPM_{i}\times \left(1-MI_{i}\right)}{HI_{i}\times \left(1-WAS_{i}\right)}$$

(4) $$CUEM_{A2}=\frac{\left(CUPM_{k0}+CUPM_{k}/\mu_{k}\right)\times \left(1-MI_{k0}\right)}{HI_{k0}\times \left(1-WAS_{k0}\right)}$$

where $CUEM_{A}$ represents the total consumption in farmland ecosystem (kg). In Formula (3), $CUEM_{A1}$ represents the ecosystem consumption needed by farmland to provide cereal, vegetable, and bean products (kg), $HI_{i}$ is the harvest index, $MI_{i}$ is moisture content (%), detailed information can be seen in Table 2, and *WAS* refers to product loss rate of 10% in this study. $CUEM_{A2}$ refers to the ecosystem consumption needed by farmland to provide sugar crops and oil crops (kg), $CUPM_{k0}$ refers to the consumption of oil or sugar crops by residents (kg), $CUPM_{k}$ refers to consumption of oil or sugar products by residents (kg), $\mu_{k}$ refers to the coefficient of converting oil or sugar into oil crops and sugar crops, and $HI_{k0}$ and $MI_{k0}$ refer to the harvest index and moisture content of the related product, respectively.

(2) Food consumption calculation in grassland ecosystem

Grassland ecosystem consumption mainly included the ecosystem consumption caused by livestock grazing and grazing and livestock providing meat, milk, and egg products (Table 1). The specific calculation formula is shown as follows [18,19]:

(5) $$CUEM_{G}=\overset{n}{\underset{i=1}{\sum }}\frac{\left(CUPM_{i}\times \theta_{i}\right)\times \left(1-MI_{i}\right)}{HI_{i}\times \left(1-WAS_{i}\right)}$$

where $CUEM_{G}\;$ refers to the ecosystem consumption required by residents to use meat and milk products provided by livestock (kg), $\theta_{i}$ refers to the coefficient of converting meat, eggs, and milk into corn, and $HI_{i}$ and $MI_{i}$ refer to the harvest index and moisture content of maize, respectively.

(3) Food consumption calculation in forest ecosystem

The consumption of forest ecosystem mainly included the consumption of ecosystems needed by forests to provide fruits (Table 1). The specific calculation formula is shown as follows [18,19]:

(6) $$CUEM_{F}=\overset{n}{\underset{i=1}{\sum }}\frac{CUPM_{i}\times \left(1-MI_{i}\right)}{HI_{i}\times \left(1-WAS\right)}$$

where $CUEM_{F}$ refers to the ecosystem consumption required by forests to provide various fruits (kg), $CUPM_{i}$ refers to the consumption of various fruit products by residents (kg), and $HI_{i}$ and $MI_{i}$ refer to the harvest index and moisture content of different fruits (Table 2), respectively.

(4) Food consumption calculation in aquatic ecosystem

The consumption of aquatic ecosystem mainly included the consumption of ecosystem needed by the aquatic area to provide fish, shrimp, crabs, and other products (Table 1). The specific calculation formula is shown as follows [18,19]:

(7) $$CUEM_{W}=\overset{n}{\underset{i=1}{\sum }}\frac{CUPM_{i}\times FCR_{i}\times \left(1-MI_{i}\right)}{1-WAS_{i}}$$

where $CUEM_{W}$ refers to the total consumption of aquatic ecosystem (kg), $CUPM_{i}$ refers to the consumption of various aquatic products by residents (kg), $FCR_{i}$ refers to the feed meat ratio coefficient of aquatic products (freshwater fish of 3, demersal fish of 6, pelagic fish of 12, marine fish of 6, shrimp of 130, and other crustacean of 150 [27]), $MI_{i}$ refers to the moisture content of aquatic products, and $WAS_{i}$ refers to the harvest loss rate of aquatic products, where $MI_{i}$ and $WAS_{i}$ refer to 75% and 10%, respectively [28,29].

## 3. Results

According to the gained data and related consumption quantity, food consumption patterns showed different characteristics in different periods. The per capita food consumption of 700 kg was taken as the dividing standard for different food consumption periods in Bangladesh, in which the food consumption pattern can be divided into three periods in combination with time change: first period from 1961 to 1971, food consumption focused on cereal–sugar–aquatic pattern; second period from 1972 to 1997, food consumption focused on cereal–sugar–aquatic–oil pattern; and third period from 1998 to 2020, food consumption pattern mainly focused on cereal–aquatic–tuber–sugar–fruit–vegetable–meat pattern. In each food consumption pattern, the consumption proportion sum of the representative foods in the total food consumption all was more than 80%.

### 3.1. Changes of Total and per Capita Food Consumption in Bangladesh

**Figure 1:**
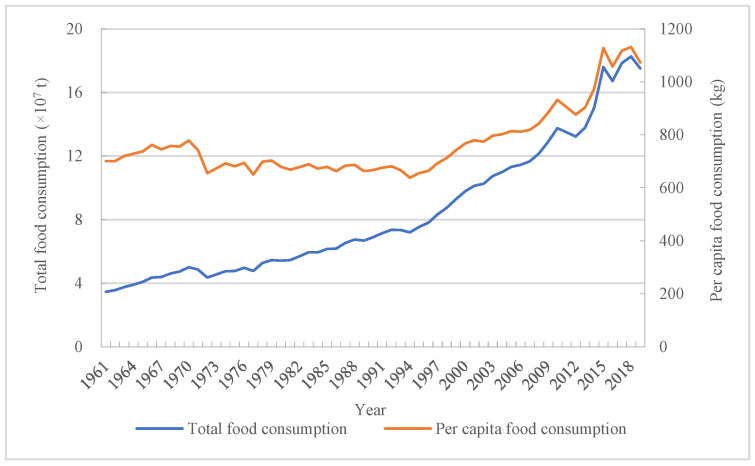
Changes of total and per capita food consumption in Bangladesh.

It was found that the total food consumption in Bangladesh showed an increasing trend on the whole, but there also was some fluctuated decline in the process. With the effect from sustained population growth, per capita food consumption experienced significant fluctuation. For total food consumption in Bangladesh, from 1961 to 2020, the least and biggest were 3.46 × 10^7^ t and 19.02 × 10^7^ t, and the biggest one had a 4.50-fold more than the least. In specific divided periods such as the first period, the average food consumption reached to 4.25 × 10^7^ t (Figure 1). In the second period, the average food consumption achieved some increase of 44.16%, which reached to 6.13 × 10^7^ t. When it came to the third period, the average food consumption reached to 13.29 × 10^7^ t, which had a growth rate of 116.70% compared to the second period’s average food consumption. The change of per capita food consumption during the research period showed significant fluctuation when compared with total food consumption’s sustained growth. Per capita food consumption only in the second period was kept below 700 kg and showed a decreasing trend. From 1961 to 1971, per capita food consumption showed some increase, although its biggest one only achieved a growth rate of 6% than in 1961. Compared with the previous two periods, per capita food consumption from 1998 to 2020 achieved a substantial increase with still significant volatility. Its biggest per capita food consumption reached to 1155.14 kg in 2020, which is 62.09% higher than the minimum value in the period.

### 3.2. Characteristics of Food Consumption in Different Ecosystems

**Figure 2:**
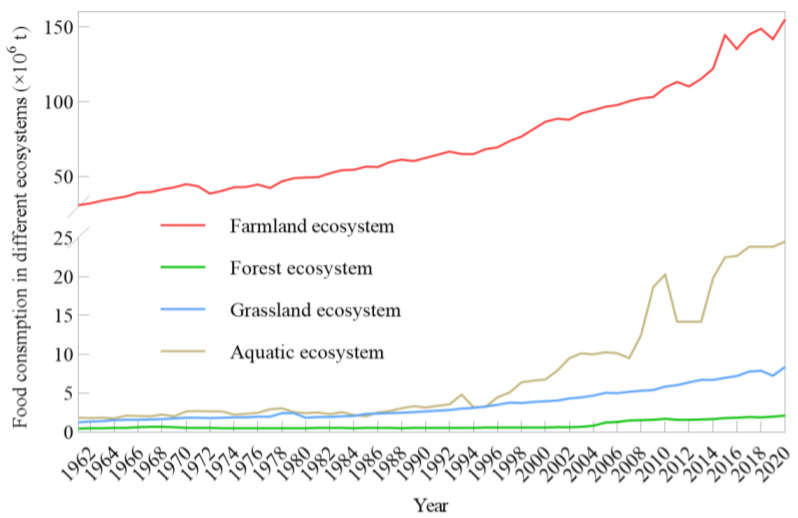
Food consumption in different ecosystems.

According to data analysis, it was found that food consumption in Bangladesh mainly came from farmland ecosystem. The proportion of food consumption of farmland ecosystem in total food consumption was kept over 80%. This was consistent with the fact that Bangladesh was a country focusing on agricultural development during the research period. The food consumption in farmland ecosystem was on the rise as a whole. Its minimum and maximum food consumption were 3.10 × 10^7^ t in 1961 and 15.52 × 10^7^ t in 2020, respectively (Figure 2). As for the other three ecosystems, the quantity of food consumption was relatively small. Food consumption in aquatic ecosystem was kept below 5 × 10^6^ t before 1997, and then it achieved a significant increase to the maximum value of 2.45 × 10^7^ t since 2017. The food consumption in aquatic ecosystem had a great increase of 12.63-fold between the maximum and minimum, and the change of food consumption presented oscillatory during the latter half of the period. The sustained increase of food consumption in aquatic ecosystem had the relationship with natives continuously developing fishery. The food consumption in forest and grassland ecosystem showed small change. Specifically, the biggest consumption in forest ecosystem did not reach to more than 2 × 10^6^ t until 2019, and the change range between the maximum and minimum was only 3.36-fold. Although food consumption in grassland ecosystem mainly increased sustainably, it achieved some decrease in 2019. The minimum and maximum food consumption in grassland ecosystem were 1.30 × 10^6^ t and 7.91 × 10^6^ t, respectively, and the maximum value had a 5.45-fold compared to the minimum value.

### 3.3. Food Consumption Patterns and Changes

**Figure 3:**
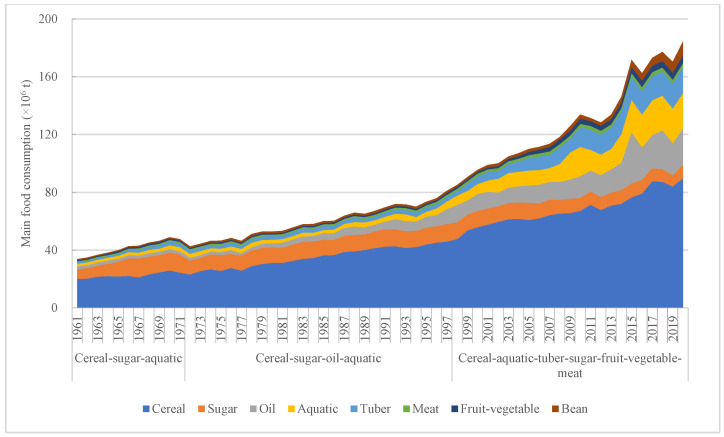
Change of main food consumption and its patterns.

The total amount of food consumption in Bangladesh increased significantly. According to food consumption quantity and composition, food consumption patterns in Bangladesh can be divided into three kinds, which were cereal–sugar–aquatic pattern, cereal–sugar–oil–aquatic, and cereal–aquatic–tuber–sugar–fruit–vegetable–meat. The proportion of food consumption quantity of each food consumption pattern reached over 80%, and the food types could represent the food consumption structure and characteristic in each related food consumption period. Although cereal consumption quantity kept growing, the proportion in total food consumption showed a decreasing trend to some extent. Its maximum proportion appeared in 1990, which reached to 60.16%, but it decreased to 43.48% in 2015 (Figure 3). Although cereal consumption proportion achieved a little rise in the following several years, it still was below 50%. Sugar played a great role in Bangladesh diet. Specifically, in food consumption pattern of cereal–sugar (from 1961 to 1971), the sugar consumption quantity presented stable growth, which increased from 6.56 × 10^6^ t in 1961 to 12.65 × 10^6^ t in 1971. The average sugar consumption proportion in this pattern was 24.35%, and the maximum was close to 30% in 1967. Apart from cereal and sugar consumption, the aquatic consumption also showed a great growth. The aquatic consumption in the cereal–sugar–aquatic pattern increased from 1.87 × 10^6^ t in 1961 to 2.74 × 10^6^ t, and its average annual growth rate was 4.24%.

When entering the cereal–sugar–oil–aquatic pattern, the oil consumption increased significantly compared with the first consumption pattern. The maximum consumption of oil had a growth rate by 4.91-fold compared with the minimum consumption. Although the cereal consumption kept growing, its increasing rate was lower than oil consumption. Additionally, the maximum cereal consumption was only 98.50% higher than the minimum cereal consumption. When entering into the third food consumption pattern, the main food types showed more diversity. In terms of changes in food consumption quantity, except for the decline in sugar consumption, other kinds of food consumption all showed an increasing trend. Specifically, for oil, aquatic, and tuber consumption in the third food consumption pattern, these three kinds of maximum food consumption got a significant growth by 1.68-fold, 2.81-fold, and 4.58-fold, respectively. Although cereal consumption quantity still increased, its proportion in the total food consumption showed an opposite trend and it had decreased to below 50%. The sugar consumption proportion in the total food consumption decreased to 4.75% in 2020.

## 4. Discussion

Based on the available data and materials, this study quantitatively discussed the evolution of food consumption in Bangladesh under the driving factors of social, economic, and ecological aspects. It was found that influencing factors such as GDP, per capita GDP, cereal production, and population had a positive effect on food consumption, while the relationship between arable land and food consumption was negative.

### 4.1. Social Factors

**Figure 4:**
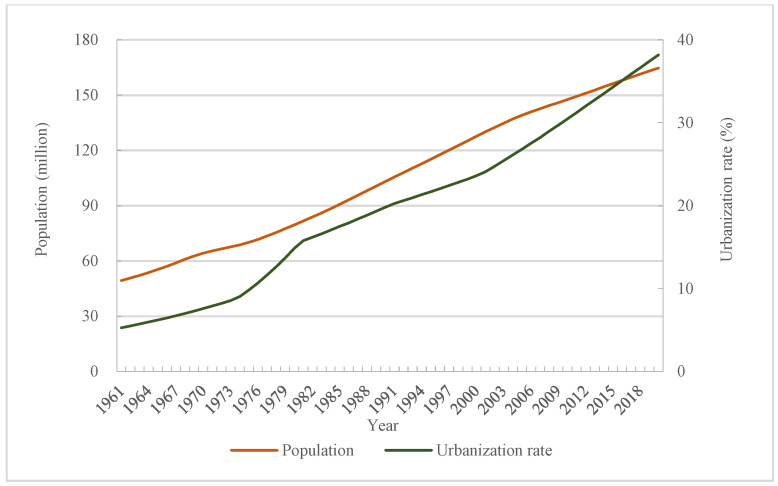
Change of population and urbanization rate.

Population increase means more pressure on food consumption, and diversified demand and challenge for food will occur in the future [30]. In this study, the population in Bangladesh kept a stable growth, which had an increase rate of 2.34-fold when comparing the maximum population in 2019 with the minimum population in 1961. At the same time, the total food consumption and per capita food consumption also achieved an increase to different extents. Specifically, the total food consumption achieved a growth rate of 4.50-fold during the whole research period. The sustainable growing population raised more demand for food. Apart from population increase, the urbanization also played a great role on demanding more food consumption [31]. With the continuous improvement of social development, the urbanization is constantly improving. Although the urbanization in Bangladesh was not as high as the developed countries, its urbanization had increased from 5.28% to 37.41% (Figure 4). This also meant that the demand for food consumption and variety was increasing. The higher urbanization requires more improved transportation facilities, which also make it possible and convenient to obtain more food. Additionally, this also provides much possibility for more food consumption to some extent.

### 4.2. Economic Factors

**Figure 5:**
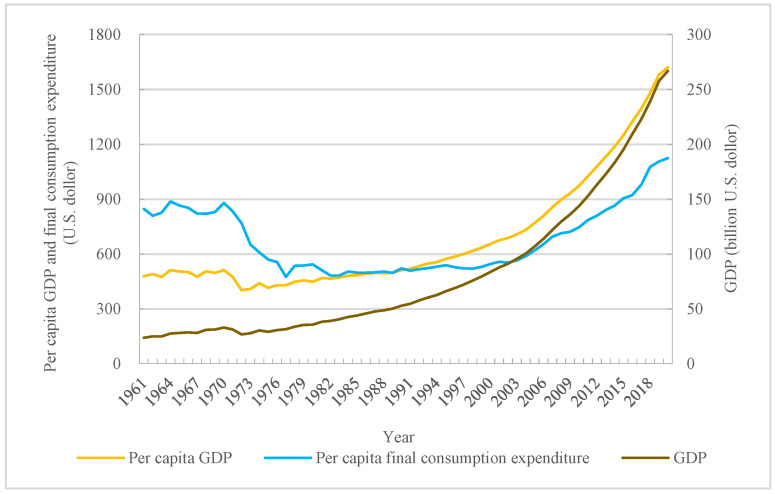
Change of per capita GDP, per capita final consumption expenditure, and GDP.

GDP represents the whole economic development and prosperity to some extent in one country or region. Additionally, it also shows the production ability of goods and services produced during a set period. This influencing factor plays a great role in residents’ purchasing power such food, especially in those areas with low economic development level [32,33]. Bangladesh is a country focusing on agricultural development, and its GDP achieved a significant improvement during the research period. Although it was only USD 23.60 billion at the beginning, it achieved a growth of more than 10-fold in the past 60 years, and it reached to USD 266.76 billion in 2020 (Figure 5). This provided a strong support for the improvement of food consumption in this country. Although GDP growth was accompanied by population growth, its per capita GDP level was still improving. Additionally, the per capita could not reflect the true household economic ability, but its annual growth could show that residents hold more choice to consume food consumption to some extent. Compared with the per capita GDP in 1961, it achieved a great growth by 2.39-fold in 2020. This meant the residents received some ability or possibility to improve food consumption.

Apart from GDP and per capita GDP, per capita final consumption expenditure reflected residents’ actual expenditure level on purchasing goods [34,35]. The change of per capita final consumption expenditure presented that it experienced a similar inverted U-shaped change process (Figure 5), and it achieved an increase by 32.72% when comparing the per capita GDP in 2020 with that in 1961. Although the increase rate was not so high as that of GDP and per capita GDP, it could play an important role in food consumption quality [36]. Additionally, this could be proved by implying a relation between per capita final consumption expenditure and food consumption change (Table 1).

### 4.3. Ecological Factors

**Figure 6:**
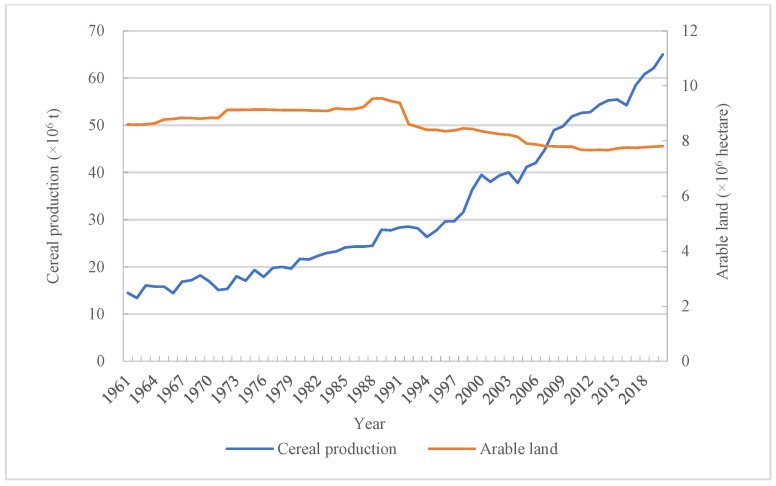
Change of cereal production and arable land.

The ecological environment and natural resources indeed provide a much significant role for the development of human beings because the basic foods are achieved from nature [37,38]. Various food production provides the solid foundation for food consumption and demand. The continuously food production could provide more food supply to meet domestic food consumption. The total food consumption could be satisfied, and the increasing food production, especially cereal production, played a great role. It was found that cereal production basically kept at a lower level which was not more than 20 × 10^6^ t before 1980. However, the cereal production had achieved a significant increase since 1980, and it reached to approximately 6.5 × 10^7^ t in 2020, which achieved a growth by 3.47-fold than that in 1961 (Figure 6). Apart from cereal production, arable land also had an effect on food consumption directly or indirectly, as enough arable land could provide foundation for continuing producing more food. However, more arable land for food did not mean more food production, as the food production not only depended on arable land but also yield level [39]. According to the correlative analysis, it was found that there was a negative relationship between arable land and food consumption (Table 3). It was undeniable that those factors played a major role on residents’ food consumption [40,41].

In summary, it was found that food consumption change was affected by many factors. The constant economic and social development in the future will stimulate more food consumption quantity and diversified food. Income and population increasing contribute to more food production and more food categories. Specifically, the diversified food provided and per capita income played a great role on food consumption choice and structure change [42,43]. This inevitably causes more land use for different food such as cereal, fruits, and vegetables [44]. All of this will take more pressure or even destroy local natural resources and ecological environment without suitable protection policies or measurements. It was necessary to formulate appropriate strategies for improving food consumption on the basis of comprehensive consideration of many factors. With the population increasing and urbanization growing, there will be a demand for more food categories and this will create more pressure on arable land use [45]. According to the results, food consumption structure became more diversified as time went on, and this meant more pressure was placed onto nature for food production [46,47]. At the same time, food consumption structure changes also equaled to the change of arable land use ways [48,49]. As was well known, the fragile natural environment and ecological condition in Bangladesh were becoming accelerated due to extensive use and lack of proper protection [50,51]. It was urgent to take effective and scientific measures to ensure the local food supply and sustainable protection of the ecological environment.

## 5. Conclusions

This study analyzed the changes in per capita food consumption and total food consumption of farmland, forest, grassland, and aquatic ecosystem in Bangladesh from 1961 to 2020. According to data and material analysis, it was found that food consumption in Bangladesh showed significant characteristics in different periods. Additionally, food consumption and structure changes were affected by a series of factors. The main results are as follows: According to food consumption structure and per capita food consumption quantity, the food consumption patterns were divided into three kinds: cereal–sugar–aquatic, cereal–sugar–oil–aquatic, and cereal–aquatic–tuber–sugar–fruit–vegetable–meat. This significantly presented that food consumption types in Bangladesh turned out to more diversified. The total food consumption kept growing. Although per capita food consumption tended to increase on the whole, it showed a certain degree of fluctuation and decline in the second food consumption pattern of cereal–sugar–oil–aquatic. Although the characteristics of food consumption from 1961 to 2020 were influenced by a series of factors, including social, economic, and ecological aspects, not every factor had a positive correlation with food consumption, such as arable land.

Although the data and material analysis in this research achieved some useful results and points, there are still some deficiencies in this study. The moisture content applied in this research was the standard values in the countries along the “Belt and Road”. Additionally, it was not specific to Bangladesh, which would have some negative effect on the final data accuracy to some extent. As for the limited budget and feasible field research, it was difficult to obtain first-hand data reflecting changes in local food consumption. It is our important research goal to apply interview questionnaires to achieve more detailed data. It is meaningful to make food consumption into ecosystem and search the change rule. This research can help us to further study the content of the relationship between resources consumption and the natural ecosystem.

## Figures and Tables

**Table 1:** Consumption category for the products provided by ecosystem.

Ecosystem Type	Specific Product
Farmland ecosystem	Cereal (rice, wheat, barley, maize, millet, and sorghum)
	Tuber (cassava, potatoes, and sweet potatoes)
	Bean (bean, pea, pulse, and soyabean)
	Sugar
	Oil (sesame, cotton, sunflower, groundnut, rape, and mustard)
	Vegetable (tomato, onion, broccoli, pepper, and other vegetables)
	Nut
Grassland ecosystem	Beef, mutton, and chicken
	Milk
	Egg
Forest ecosystem	Fruit (orange, lemon, lime, apple, banana, mongo, pineapple, and grapefruit)
Aquatic ecosystem	Fish (freshwater fish, demersal fish, pelagic fish, and marine fish)
	Shrimp (shrimp, brown shrimp, and lobster)
	Crustacean

**Table 2:** The harvest index and moisture content of different food [18,19].

Food Type	Harvest Index	Moisture Content (%)	Food Type	Harvest Index	Moisture Content (%)
Rice	0.50	13	Other tuber	0.67	13.3
Maize	0.49	13	Rapeseed	0.26	9
Wheat	0.46	13	Sesame	0.34	9
Millet	0.31	13	Groundnut	0.50	9
Other cereal	0.38	13	Other oilseed	0.36	9
Sorghum	0.31	13	Sugar crop	0.71	13.3
Barley	0.49	13	Sugar	0.7	13.3
Bean	0.42	13	Vegetable	0.49	82
Potato	0.59	13.3	Fruit	0.49	82

**Table 3:** Correlations between per capita food consumption and different influencing factors.

Influencing Factors	Correlation Coefficient (*r*)	*p* Value	Period of Data
Population	0.716 **	<0.01	1961–2020
Urbanization rate	0.739 **	<0.01	1961–2020
Cereal production	0.856 **	<0.01	1961–2020
Arable land	−0.781 **	<0.01	1961–2020
Per capita GDP	0.958 **	<0.01	1961–2020
Per capita final consumption	0.768 **	<0.01	1961–2020
GDP	0.931 **	<0.01	1961–2020

** represents significance at 1% level.

## Data Availability

Data are contained within the article.
